# NIGHTTIME AMBIENT TEMPERATURE AND SLEEP IN OLDER ADULTS

**DOI:** 10.1093/geroni/igae098.0618

**Published:** 2024-12-31

**Authors:** Amir Baniassadi, Wanting Yu, Thomas Travison, Lewis Lipsitz, Brad Manor

**Affiliations:** Harvard Medical School/Hebrew SeniorLife, Boston, Massachusetts, United States; Hebrew SeniorLife, Boston, Massachusetts, United States; Harvard Medical School/Hebrew SeniorLife, Boston, Massachusetts, United States; Hebrew SeniorLife, Boston, Massachusetts, United States; Harvard Medical School/Hebrew SeniorLife, Boston, Massachusetts, United States

## Abstract

**Background:**

Sleep quality is a key contributor to overall health and wellbeing in older adults. While lab-based studies demonstrate that changes in ambient temperature influence sleep quality and duration, our understanding of these associations in older adults’ own home, where they have some degree of control over the temperature, is limited.

**Aim:**

To examine the relationships between bedroom ambient temperature and outcomes related to sleep in community dwelling older adults.

**Methods:**

In this longitudinal observational study, we monitored 50 older adults (average age=79 ±7.2y, 41 females) living in Boston, for 18 months. We used wearable sleep monitors and environmental sensors to measure sleep duration, and efficiency (ratio of time spent asleep to time spent in bed) along with bedroom temperature and humidity.

**Results:**

Sleep was most efficient when the nighttime ambient temperature was between 20-24˚C, with a clinically relevant 5-10% drop in sleep efficiency when the temperature increased beyond this range by 5 ˚C. The associations were primarily nonlinear, with significant between-subject variations.

**Discussion:**

Sleep disturbances and difficulties falling asleep are common in older adults, often without undiagnosed underlying conditions. Our study identifies bedroom temperature as a crucial regulator of sleep even within home where the individual can control the temperature. This highlights the potential impact of climate change on sleep in older adults, especially those of lower socioeconomic status. Furthermore, our findings suggest that optimizing bedroom temperature can enhance sleep quality in older adults and emphasize the importance of personalized temperature adjustments based on individual needs and circumstances.

